# Cardiac metastasis from small cell lung cancer origin: A case report and review of the literature

**DOI:** 10.1002/cnr2.1711

**Published:** 2022-09-18

**Authors:** Lei Tian, Miaomiao Liu, Liya He, Qi Zhang, Qiaofang Li, Hongzhen Zhang

**Affiliations:** ^1^ The Fifth Department of Oncology Hebei General Hospital Shijiazhuang Hebei China

**Keywords:** cardiac magnetic resonance, metastatic cardiac tumors, small cell lung cancer

## Abstract

**Background:**

Cardiac metastasis from small cell lung cancer (SCLC) origin is rare, whereas the incidence is anticipated to increase with the extended survival rates. Case: We here describe a case report of a 48‐year‐old male patient diagnosis with SCLC in 2020. In June 2021, he resorted to hospital due to shortness of breath, no obvious changes were found in repeated echocardiography, electrocardiogram and chest computer tomography from June 2021 to September 2021. Due to the persistence of the complaints, cardiac magnetic resonance (CMR) imaging was performed in September 30th, 2021, which showed a mass in the right atrioventricular groove. The patient underwent pericardiocentesis and small cell carcinoma cells were found in the pericardial effusion, confirming the diagnosis of cardiac metastasis.

**Conclusion:**

Patients with a history of SCLC who develop new cardiac symptoms of unknown etiology should undergo imaging studies such as CMR. The importance of CMR for patients with SCLC is highlighted. The literature regarding metastatic cardiac tumors is reviewed.

## 1 INTRODUCTION

Primary malignant cardiac tumors are rare with an incidence of 0.009% while metastatic cardiac tumors (MCT) are more common at 1.8%.^1^, ^2^, ^3^, ^4^ Whereas any type of cancer may spread to the heart, the most frequent origins of MCTs are malignant melanoma and leukemia.^4^ Cardiac metastases arising from breast, head and neck and colon cancer have been reviewed elsewhere.^5^, ^6^, ^7^ However, cardiac metastasis from SCLC is often symptomless and rarely diagnosed until cancer cells appear in the pericardial effusion.

## 2 CASE

A 48‐year‐old male was referred to our department (July 2, 2020) complaining of cough and expectoration during the previous 4 days. Computer tomography (CT) of the chest showed a left perihilar lesion measuring approximately 93 × 59 × 76 mm which had metastasized to the upper left pulmonary lobe and the adrenal gland. Bronchoscopy and pathological biopsy revealed small cell lung cancer (SCLC) with positive immunohistochemical results for Syn, CgA, and CD56. The cancer was staged as SCLC, cT4N0M1 stage IV. Radiation therapy was given to the left lung and adrenal gland, in combination with chemotherapy (Etoposide plus Cisplatin) and immunotherapy (Durvalumab). A second CT (November 20, 2020) scan following 6 cycles of chemotherapy and immunotherapy revealed that the lesions of the lung and adrenal gland had shrunk and then received immune maintenance therapy.

**FIGURE 1:**
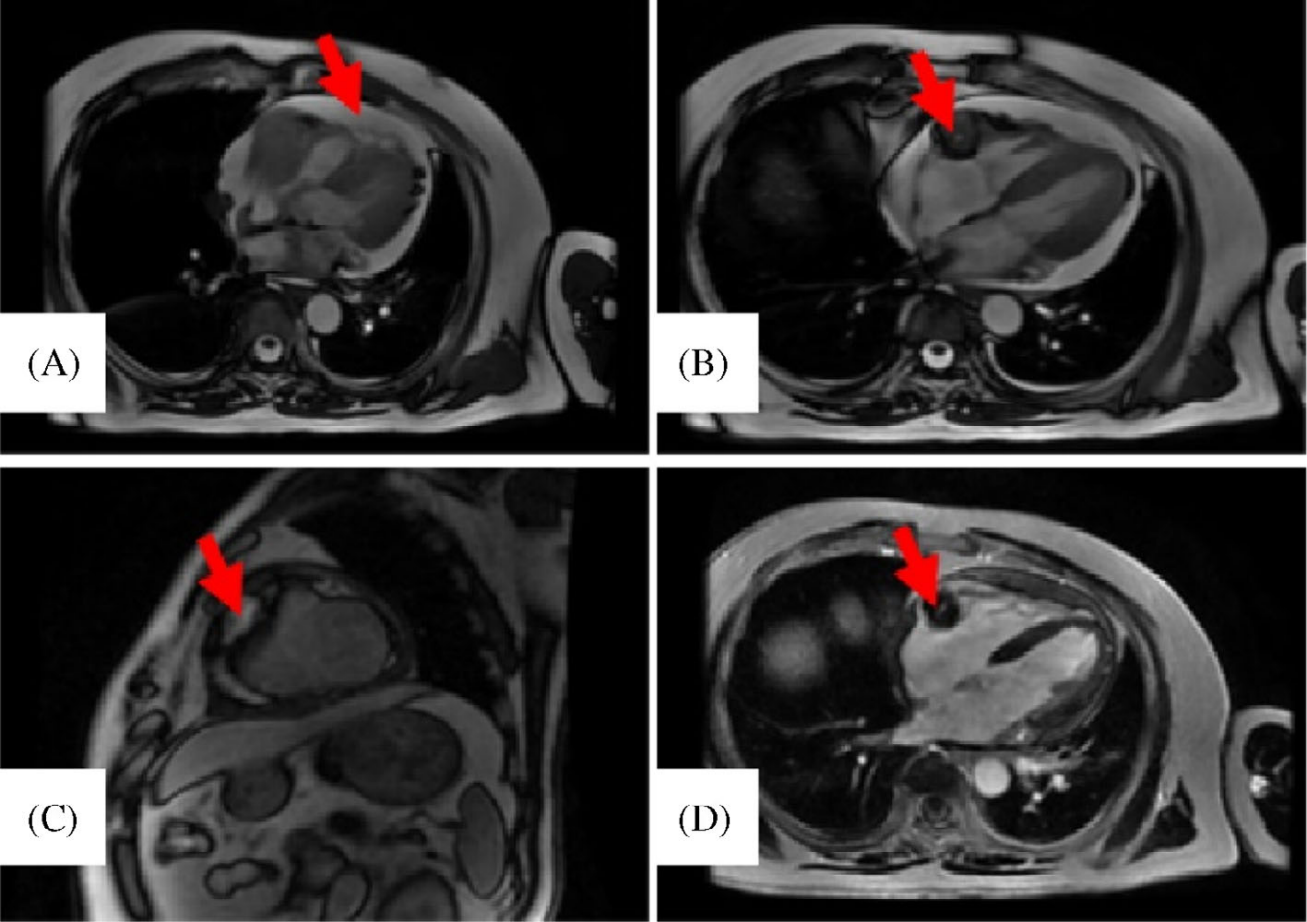
Imaging revealed cardiac metastases. (a) A small amount of pericardial effusion was detected. (b) Cardiac magnetic resonance imaging showed a mass (36 mm × 24 mm × 54 mm) with higher signal intensity on T2 sequences, invading the right coronary arteries and pericardium. (c) The mass showed markedly enhanced signal in myocardial resting perfusion imaging. (d) The mass had hypointense in delayed enhanced imaging.

He resorted to hospital due to shortness of breath in June 2021. PET/CT examinations revealed stable disease. From June 2021 to September 2021, he was given repeated echocardiography, electrocardiography (ECG), and chest CT and no obvious changes were found. Due to the persistence of the complaints, cardiac magnetic resonance (CMR) imaging was performed in September 30, 2021, which showed a small amount of pericardial effusion and a mass (36 mm × 24 mm × 54 mm) in the right atrioventricular groove. It is hyperintense on T2 sequences with infiltration of the right coronary arteries and pericardium. In myocardial resting perfusion imaging, the mass showed markedly enhanced signal while the mass had hypointense in delayed enhanced imaging (Figure 1). Later echocardiography examination (in October 2021) revealed a hypoechoic mass of about 36.8 mm × 51.3 mm × 31.7 mm in size in the pericardial cavity of the right atrioventricular groove, this is consistent to the findings of CMR. ECG was normal. A cardiac metastasis was suspected but the patient's symptoms were tolerable and complete removal of the mass would have been difficult. The patient was treated with Durvalumab and Anlotinib Hydrochloride Capsules.

More than 2 months later (December 6, 2021), the patient was hospitalized owing to worsening shortness of breath and difficulty breathing. Heart sounds were distant and muffled on physical examination. Echocardiography demonstrated massive pericardial effusion and pericardiocentesis significantly relieved the symptoms. Small cell carcinoma cells were found in the pericardial effusion, confirming the diagnosis of cardiac metastasis. The patient received intravenous albumin‐bound paclitaxel combined with intrapericardial cisplatin and died from a general deterioration of his condition on March 1, 2022.

## 3 DISCUSSION

Around 75%–90% of primary cardiac tumors are benign,^1^, ^3^, ^8^, ^9^ while MCTs occur at 20 times the frequency of primary cardiac tumors.^8^, ^9^ Improved diagnostic and therapeutic modalities have led to increased identification of MCT. Tumors can spread to the heart via direct extension, the lymphatic system, hematogenous, or transvenous routes, especially through the inferior vena cava.^2^, ^5^

Clinical manifestations of MCT vary widely and include arrhythmias, embolic phenomena or acute coronary syndrome.^8^, ^9^, ^10^ Patients may also experience non‐specific manifestations, such as fever, malaise and arthralgia. Shortness of breath is the most common symptom and cardiac tamponade is life threatening. In the present case, the patient's main complaint was shortness of breath, especially during activity.

The diagnosis of MCT is often fortuitous or delayed, due to the non‐specific nature of the symptoms. The diagnostic value of ECG is limited and tissue histopathology remains the gold standard. Due to the clinical inaccessibility of tissue samples for histopathology, transthoracic echocardiography (TTE) is the most common non‐invasive, low‐cost diagnostic method, showing high sensitivity (90%) and specificity (95%).^4^, ^11^ However, TTE alone is not sufficient for obtaining more detailed information, especially their relationships with adjacent tissue, while tumors usually infiltrate adjacent tissue. Transesophageal echocardiography (TEE) provides more clear and precise information about cardiovascular morphology and anatomy and is an effective complement to TTE.^12^ Three‐dimensional (3D) TEE has been suggested to be able of producing high‐quality images of cardiac tumor as it offers real‐time images and their relationships with adjacent organs. Fontana et al. suggested that 3D TTE has an improved value in the diagnosis of cardiac metastasis from malignant melanoma.^13^

Although echocardiography is excellent in the diagnosis of MCT, small field of view and insufficient acoustic window limit its application. Sometimes echocardiographic findings is normal in patients with cardiac metastases.^14^ While CMR is the gold standard for the definition of the nature of intracardiac masses and their relationship to paracardiac structures.^15^ CMR allows optimal tumor characterization with superior depiction of soft tissues and provides accurate evaluation of cardiac structure and function to complement echocardiography and provide early detection and management.^16^, ^17^ The benefits of CMR over echocardiography in studying cardiac tumors has been recognized.^14^ Previous study suggested that cardiac metastases can be mistaken for thrombi, endocarditis, or primary tumors in echocardiograms, supplemental imaging with CMR is often needed for further characterization.^18^, ^19^ Though several studies have confirmed the value of 18F‐FDG PET/CT,^20^, ^21^ it has not yet achieved the status of a routine examination method to evaluate cardiac metastasis, perhaps due to high FDG uptake by the myocardium.^22^ In the current case, the mass was first detected by CMR, whereas no abnormality was found in previous repeated ultrasonography. This once again verified that CMR is superior to ultrasonography, which may have a lag in diagnosing cardiac metastases.

The low incidence of MCT means that there are, as yet, no evidence‐based guidelines for optimal treatment.^2^ The most suitable strategy is decided bases on symptoms. For patients presenting with symptoms of cardiac tamponade, pericardiocentesis is necessary. Surgical resection of metastases in the heart not only relieves the symptoms, but also leads to a favorable prognosis.^5^ Heart transplantation may be successful in appropriate patients.^3^ However, systemic therapy is the definitive treatment since patients with cardiac metastases often present with a great tumor burden.^9^

The prognosis depends on many factors and, despite advances, remains poor for MCT patients. Previous studies have shown a median survival ranging from 1 to 18 months.^6^, ^21^ The current patient survived for 5 months following pericardiocentesis plus systemic therapy.

In summary, cardiac metastasis from a SCLC origin is uncommon and has a poor prognosis. Patients can be asymptomatic or suffer life‐threatening symptoms. In our patient, CMR correctly identified the location of the metastases, as well as its size and relationship to right coronary arteries and pericardium. This report reaffirms the superiority of CMR over other imaging modalities in the diagnosis of cardiac metastases.

## AUTHOR CONTRIBUTIONS

**Lei Tian:** Conceptualization (lead); data curation (supporting); formal analysis (equal); funding acquisition (lead); investigation (equal); methodology (lead); project administration (equal); resources (equal); software (lead); supervision (equal); validation (supporting); visualization (equal); writing – original draft (lead); writing – review and editing (lead). **Miaomiao Liu:** Conceptualization (equal); data curation (lead); formal analysis (lead); funding acquisition (supporting); investigation (lead); methodology (supporting); project administration (supporting); resources (supporting); software (equal); supervision (supporting); validation (supporting); visualization (supporting); writing – original draft (supporting); writing – review and editing (equal). **Liya He:** Conceptualization (supporting); data curation (equal); formal analysis (equal); funding acquisition (supporting); investigation (supporting); methodology (supporting); project administration (lead); resources (equal); software (supporting); supervision (supporting); validation (supporting); visualization (supporting); writing – original draft (supporting); writing – review and editing (supporting). **Qi Zhang:** Conceptualization (supporting); data curation (equal); formal analysis (supporting); funding acquisition (supporting); investigation (supporting); methodology (supporting); project administration (supporting); resources (supporting); software (supporting); supervision (supporting); validation (supporting); visualization (supporting); writing – original draft (supporting); writing – review and editing (supporting). **Qiaofang Li:** Conceptualization (supporting); data curation (supporting); formal analysis (supporting); funding acquisition (supporting); investigation (supporting); methodology (supporting); project administration (supporting); resources (supporting); software (supporting); supervision (supporting); validation (supporting); visualization (supporting); writing – original draft (supporting); writing – review and editing (supporting).

## FUNDING INFORMATION

Health Commission of Hebei Province, Grant/Award Number: 20220919.

## CONFLICT OF INTEREST

The authors have stated explicitly that there are no conflicts of interest in connection with this article.

## ETHICS STATEMENT

The authors declare that the work described was original research that has not been published previously, and not under consideration for publication elsewhere, in whole or in part. All the authors listed have approved the manuscript that is enclosed and agreed to the terms of the Wiley Open Access agreement and pay the associated fee.

## Data Availability

The data that support the findings of this study are available from the corresponding author upon reasonable request.
